# Beneficial arthropods threatened by synthetic agricultural pesticides around the southeast slopes of Mount Cameroon

**DOI:** 10.1016/j.heliyon.2025.e42241

**Published:** 2025-01-27

**Authors:** Daniel Brice Nkontcheu Kenko, Agathe Lambou Fotio, Kenfack Donhachi Aimerance, Derick Atemlefac Acha, Miranda Egbe Awo, Eric Bertrand Fokam

**Affiliations:** a Department of Animal Biology and Conservation, Faculty of Science, University of Buea, Cameroon; b Biology and Applied Ecology Research Unit, University of Dschang, Cameroon; c Advanced School of Agriculture, Forestry, Water Resources and Environment, University of Ebolowa, Cameroon; d Applied Hydrobiology and Ichthyology Research Unit, University of Dschang, Cameroon; e Department of Plant Science, Faculty of Science, University of Buea, Cameroon

**Keywords:** Pollinators, Bees, Non-target arthropods, Pesticide risk assessment, Mount Cameroon, Agriculture

## Abstract

Agriculture is among the main drivers of global biodiversity decline. This research assessed the ecological risk of synthetic pesticides at the southeast slopes of Mount Cameroon on bees and other non-target arthropods (NTAs) using the PRIMET (Pesticide RIsk in the tropics to Man, Environment and Trade) model. Data on pesticide usage (active ingredients, interval, dosage, number of applications), and ecotoxicology (LD_50_ and environmental behaviour) were collected from field surveys and the IUPAC databases, respectively, then imported into PRIMET to assess the level of risk expressed in Exposure Toxicity Ratio (ETR). Tomato was the main crop of the area while insecticides were the main pesticide category utilised in the study area. Nine (09) compounds were predicted to pose a “risk” to bees namely abamectin, fipronil, imidacloprid, thiamethoxam, emamectin-benzoate, cypermethrin, lambda-cyhalothrin, indoxacarb and ethoprophos. Abamectin had the highest ETR in the group (NEC_bee_ = 0.05 g a.i./ha; ETR_bee_ = 20000). Fifteen (15) active ingredients were predicted by the model to pose a certain in-field risk to NTAs with lambda-cyhalothrin at the top position (AEC_NTA_ = 0.0034 g a.i./ha; ETR_IF_ = 1147000). There was evidence that insecticides were of more risk to beneficial arthropods, as compared to other types of pesticides. Pesticides active ingredients such as abamectin, fipronil, imidacloprid, thiamethoxam, emamectin-benzoate, cypermethrin and lambda-cyhalothrin that were predicted to pose “Risk” both to bees and others NTAs require serious mitigation strategies (biological control, improve cultural practices, target application, farmer's education).

## 1 Introduction

Due to is cultural, ethnic, and geographical diversity, Cameroon is commonly known as Africa in miniature. The country has a unique worldwide biodiversity hotspot distributed especially along the Cameroon volcanic line, which ranges from the Bioko Island to Lac Chad. In this rich biodiversity arthropods occupy a tremendous position because of their number, diversity as well as interactions with humans. Bees form a tremendous component of biodiversity, providing key ecosystem services: production of many valuable products (honey, bee wax) and pollinators of many crops [1,2]. Crop pollination isa key factor that boosters economy and is carried out by bees (honey bees, bumble bee, stingless bee, carpenter bees, solitary bees) and other insects (hoverflies, butterflies, moths, beetles, thrips and wasps) for a variety of plants [3]. Arthropods in general are very helpful as pest control agents (predators and parasitoids) and scavengers; nonetheless, some arthropod species are agricultural pests. Both groups are important components of the biodiversity and terrestrial food webs. Non-target arthropods are represented by *Aphidius rhopalosiphi* (Hymenoptera: Braconidae) and *Typhlodromus pyri* (Acarina: Phytoseiidae), very valuable (parasitoids and predatory, respectively) susceptible arthropods [4]; their adoption as arthropod models is related to their sensitivity as taking care of them will safeguard many other organisms.

Nevertheless, the global biodiversity decline is currently a major concern in science. Several factors account for this decline including habitat destruction and fragmentation, interspecific and intraspecific competition, overexploitation, invasive and exotic species, climate change, parasites and diseases, pollution, and poor regulation on agrochemicals [[5], [6], [7]], Among these drivers of biodiversity decline, pollution represent a main contributor. Environmental pollutants have many sources, industrial wastes, urban and municipal wastes, and agricultural wastes. In the Cameroonian context, economic factors push more and more people into agriculture and the government is promoting second-generation agriculture. Cameroon is the food basket of the Central African sub-region. Several crops are cultivated in Cameroon by means of small-scale farming systems or agroindustries, among others, the CDC (Cameroon Development Corporation), PHP (Plantations du Haut Penja), SBM (Société des Bananerais de la Mbomé), SPNP (Société des Plantations Nouvelles de Penja), CTE (Cameroon Tea Estates), SODECOTON (Société de Développement du Coton), UNVDA (Upper Noun Valley Development Authority), SOCAPALM (Société Camerounaise de Palmerais) [8]. Pesticides are increasingly used by farmers to control pest and disease occurrence in farms. Modern agriculture is pesticide-dependent and many tons of synthetic pesticides are produced and used yearly worldwide [9]. Cameroon like most developing countries is experiencing serious issues with pesticide usage as most of the farmers exhibit very poor usage routines such as non-respect of prescribed doses, usage of illegal and banned pesticides, and no protective equipment [[10], [11], [12], [13], [14], [15]].

From available literature, the toxicity data of many pesticides is well known and documented but the LD_50_ bee and the LR_50_ NTAs alone are not enough to predict the risk related to the use of a particular compound; other variables must be considered. The actual application plan as practiced by farmers and pesticide environmental behaviour are important covariates to assess risk, hence the merits of the PRIMET model, capable of combining all these variables to model the risk associated with the usage of each pesticide. Having an international reputation, PRIMET [4] is one of the risk assessment models developed by scientists of the Wageningen University and Research Centre (WUR), the National Institute of Public Health and the Environment (RIVM) and the Netherlands Environmental Assessment Agency (PBL). Non-target organisms are highly exposed to pesticides as agricultural activities are widespread in the mono-modal equatorial agroecological zone in Cameroon because of fertile soils, yet studies documenting the pesticide effects on non-target arthropods are not very abundant apart from a few studies in the Tiko plain, which have reported pesticide risks on bees and non-target arthropods [8,16] and another studies in the Buea municipality on pesticide effects on insect visitors of four common tropical crops [17]. To contribute to research in pesticide ecotoxicology and fill the knowledge gap and contribute to pesticide ecotoxicology especially modelling, this research aimed at assessing the ecological risk of synthetic pesticides at the southeast slopes of Mount Cameroon on non-target arthropods utilising the *Pesticide RIsk in the tropics to Man, Environment and Trade* (PRIMET) model. The output of this research is to raise awareness on the implications of pesticide usage for better reinforcement of regulation and improved pesticide usage schemes, to protect human and environmental health.

## 2 Material and methods

### 2.1 Study zone

**Fig. 1:**
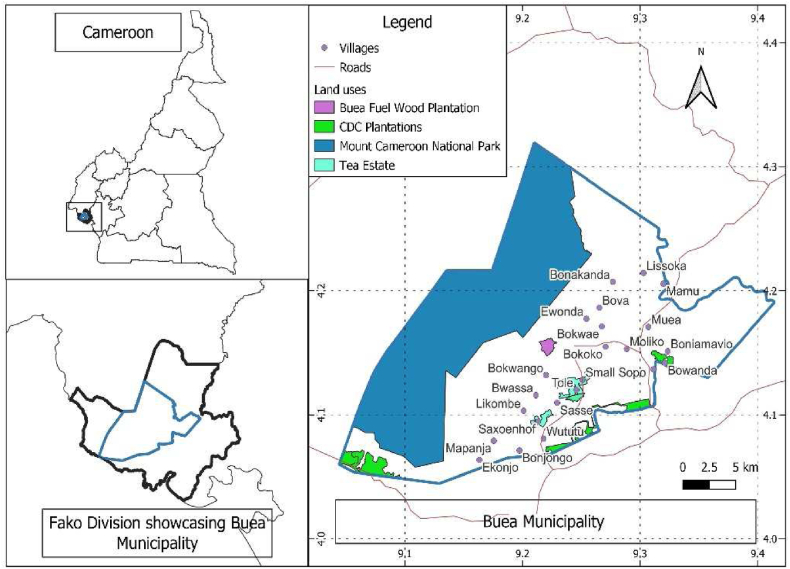
Map of the study area, Source: [48].

This research was done on the Southeastern slopes of Mount Cameroon (Buea Municipality). With an altitude of about 900 m, Buea (Fig. 1), the regional capital of the Southwest Region of Cameroon, is a historic town located at the foot and eastern slopes of Mount Cameroon, the highest mountain in West and Central Africa. The region covers approximately 2,700 km^2^ encompassing the main massif which is about half the total area of 1,500 km^2^ and summit at 4095m above sea level [18].

The mountain has a strong influence on climate causing considerable orographic rainfall, especially on the rain-bearing windward western side of the mountain where mean annual rainfall is maximum, reaching 10,000 mm in Debundscha and slowly decreasing in an easterly direction to less than 2000 mm. Temperature varies more on the west slopes (24–28 °C) than on the east slopes (27–28 °C) [18,19].

The Buea municipality is part the monomodal agroecological zone of Cameroon. This area is characterised by rich volcanic soils, with an equatorial climate conducive for agriculture, the major industry in Buea. Agriculture is carried out by large agro-industrial complexes like the CDC, CTE and PALMOL, that produce oil palm, rubber, tea and banana, as well as small-scale farmers cultivating maize, cassava, tomato, cocoa, plantain, and many vegetable species for home consumption as well as for raising incomes. Products issued from this activity supply both the local market and neighbouring countries like Gabon and Equatorial Guinea.

### 2.2 Assessment of the terrestrial risks of pesticides on bees and NTAs using PRIMET

The PRIMET model (Source: https://www.pesticidemodels.eu/primet/home) requires two (02) main groups of inputs variables for risks assessment: actual pesticide utilisation patterns as practiced by farmers, pesticide ecotoxicological properties (toxicity data).

#### 2.2.1 Determination of pesticide use patterns and ecotoxicological data

The target population in this study was small holder farmers involved in crop production. (Inclusion criteria: being a farmer, with a farm in the municipality, using pesticides in the farm and being ready to participate in the study) or owners of pesticide shops in the study area Fulfilling the farmers criteria or not).Consent to participate was first obtained from each respondent before questionnaire administration or interview Pesticide dealers were selected in main markets and shops of the municipality to form a representative sample (Buea Town, Clerk's Quarter, OIC Market, Molyko and Mua). The main agricultural spots of the town were targeted: Muea, Sand Pit, Buea Town, Mile 16, Small Soppo and Bokwango. The convenience sampling [20] strategy was adopted. Based on the above criteria seventy-five (75) semi-structured questionnaires were administered in the study area, i.e. sixty (60) to farmers and fifteen (15) to pesticide dealers.

The following information were requested from respondents: pesticide trade names, active ingredients, application rate (gram of active ingredient per hectare), number of pesticide sprays per cropping season and interval between pesticide sprays (days), for fungicides, insecticides and other pesticides (Table 1, Table 2, Table 3 respectively).

**Table 1:** Insecticides application scheme in the study site and ecotoxicological properties on bees and NTAs.

Insecticide	Crops	Interval application (days)^a^	Rate (g a.i./ha)^a^	Number of applications per crop cycle^a^	LD-_50_ bees (μg/bee)^b^	LR-_50_ NTA (g a.i./ha)^b^
Acetamiprid	Tomato	6	1000	5	8.09	2
	Huckleberry	10	900	4		
Chlorpyrifos-ethyl	Eggplant	14	1050	3	0.068	0.2
	Pepper	21	1200	4		
Cypermethrin	Maize	14	600	3	0.023	0.0029
	Cucumber	14	900	3		
	Plantain	30	1000	3		
	Tomato	14	1000	4		
	Huckleberry	21	1000	6		
Emamectin-benzoate	Sweet bitter leaf	90	350	1	0.0036	0.5
	Cassava	90	900	2		
	Sugarcane	30	500	10		
Fipronil	Plantain	30	1250	5	0.0059	0.01
	Garden egg	10	800	6		
	Cabbage	10	900	4		
	Pepper	10	700	4		
	Cassava	30	1406	4		
	Watermelon	14	700	3		
	Okra	14	2500	2		
	Maize	30	2000	2		
	Sugarcane	60	1200	5		
	Cocoa	14	1875	5		
	Tomato	14	400	6		
Imidacloprid	Huckleberry	14	500	2	0.0037	0.022
	Tomato	6	1400	8		
	Cocoa	30	900	6		
	Pepper	14	1000	3		
Indoxacarb	Huckleberry	10	900	4	0.08	5.1
Lambda-cyhalothrin	Huckleberry	21	1000	6	0.038	0.0017
	Tomato	14	1300	5		
	Maize	14	700	2		
	Pepper	14	1000	3		
Oxamyl	Beans	80	4000	2	0.38	0.03
	Tomato	30	500	1		
	Sweet bitter leaf	90	4000	1		
	Huckleberry	45	4000	2		
	Watermelon	6	600	2		
Thiamethoxam	Cocoa	60	630	3	0.005	0.13
	Cabbage	14	500	3		
	Maize	14	900	4		
	Tomato	6	1500	3		

LD_50-Bee:_ Concentration that kills 50 % of bees (μg/bee), the most sensitive endpoint of oral LD_50_ or contact LD_50_.

LR_50_NTA:_ Rate that kills 50 % of *Typhlodromus pyri* (predatory mite) or *Aphidius rhopalosiphi* (parasitic wasp), the most sensitive endpoint of the two organisms.

a Obtained from surveys.

b Obtained the PPDB.

**Table 2:** Fungicides application scheme in the study area and ecotoxicological data for bees and NTAs.

Fungicide	Crops	Interval application (days)^a^	Rate (g a.i./ha)^a^	Number of applications per crop cycle^a^	LD_50_Bees_ (μg/bee)^b^	LR_50_NTA_ (g a.i./ha)^b^
Carbendazim	Maize	14	466	4	50	30
	Tomato	7	2400	4		
Chlorothalonil	Tomato	7	2400	4	101	262
Copper oxide	Cocoa	18	950	6	22	26 100
	Tomato	14	4000	3		
Mancozeb	Garden egg	14	4000	6	85.3	21.47
	Tomato	10	2500	6		
	Watermelon	21	2600	3		
	Pepper	7	4000	10		
	Cucumber	6	4000	5		
Mefenoxam (Metalaxyl-M)	Cocoa	30	600	6	97.3	∗
	Cabbage	14	6000	4		
	Cucumber	3	4000	14		
	Cocoa	14	1000	5		
Thiram	Eggplant	14	1312	3	100	511

LD_50-Bee:_ concentration that kills 50 % of bees (μg/bee), the most sensitive endpoint of oral LD_50_ or contact LD_50_.

LR_50_NTA:_ Rate that kills 50 % of *Typhlodromus pyri* (predatory mite) or *Aphidius rhopalosiphi* (parasitic wasp), the most sensitive endpoint of the two organisms.

a Obtained from surveys.

b Obtained the PPDB.

**Table 3:** Herbicides and other pesticides application scheme in the study area and ecotoxicological data for bees and NTAs.

Pesticide	Crops	Interval application (days)^a^	Rate (g a.i./ha)^a^	Number of applications per crop cycle^a^	LD_50_Bee_ (μg/bee)^b^	LR_50_NTA_ (g a.i./ha)^b^
Herbicide						
2,4 - D	Weeds	45	40 600	2	94	3000
Paraquat	Weeds	90	2000	1	9.06	10
	Weeds	60	6000	2		
Diuron	Weeds	60	3000	1	86.75	∗
Glyphosate	Weeds	60	9000	1	100	4320
Nicosulfuron	Weeds	30	10 800	2	22.4	60
Paraquat dichloride	Weeds	60	7000	1	72	∗
Acaricide						
Abamectin	Tomato	6	1000	8	0.001	0.09
	Beans	80	1000	1		
Rodenticide						
Brodifacoum	Maize, Cassava, Plantain	90	3800	2	∗	∗
	Tomato	90	3500	2		
Nematicide						
Ethoprophos	Plantain	40	3000	4	4.07	∗
	Banana	30	32000	5		
Molluscicide						
Metaldehyde	Okra	14	9500	4	87.5	350
	Huckleberry	14	100	2		
	Plantain	30	13500	3		
	Watermelon	30	1100	3		

LD_50-Bee:_ concentration that kills 50 % of bees (μg/bee), the most sensitive endpoint of oral LD_50_ or contact LD_50_.

LR_50_NTA:_ Rate that kills 50 % of *Typhlodromus pyri* (predatory mite) or *Aphidius rhopalosiphi* (parasitic wasp), the most sensitive endpoint of the two organisms.

a Obtained from surveys.

b Obtained the PPDB.

Information on the ecotoxicology of pesticides (LD-_50_ Bees, LR-_50_ NTA) was obtained from two main IUPAC databases: Pesticide Properties Data Base (PPDB) (http://sitem.herts.ac.uk/aeru/ppdb/en) and Bio Pesticides Data Base (BPDB) (http://sitem.herts.ac.uk/aeru/bpdb/Reports/1326.htm) [21] as presented in Table 1 (Insecticides), Table 2 (Fungicides) and Table 3 (Other pesticides).

#### 2.2.2 Risk assessment on bees using PRIMET

Information from Table 1, Table 2, Table 3 was keyed into the PRIMET model, except the column LR_50_NTA_ (g a.i./ha). For each active ingredient, the model estimated the Predicted Exposure Rate (PEC), No Effect Rate, (NEC), and the Exposure Toxicity Ratio (ETR).

##### 2.2.2.1 Predicted exposure concentration (PEC_bee_)

The exposure is established as the maximum single application rate expressed as gram active ingredient per hectare (Equation (1)).

(1) $$\mathrm{PE}C_{\mathrm{bee}=}\;\mathrm{Applied}\;\mathrm{Rate}\;\left(g\;a.i.\;/\;\mathrm{ha}\right)$$

##### 2.2.2.2 No effect concentration (NEC_bee_)

For the effect assessment, a “safe” concentration was calculated from the toxicity values and an assessment correction factor (to convert from μg/bee to g a.i./ha) (Equation (2)).

(2) $$\mathrm{NE}C_{\mathrm{bee}}=EF_{\mathrm{bee}}\;x\;LD_{50\_\mathrm{bee}}$$

With,

- NEC_bee_ = No Effect Rate for bees (g a.i./ha)
- LD_50_Bee_ = Dose (oral or contact) that kills 50 % of bees (μg/bee), the most sensitive endpoint of oral LD_50_ and contact LD_50_.
- EF_bee_ = Extrapolation correction factor for effect assessment of bees, to convert from μg/bee to g a.i./ha (default value = 50).

##### 2.2.2.3 Risk to bees (ETR_bee)

The risk, expressed in the Exposure Toxicity Ratio (ETR) because of the application is computed according to Equation (3):

(3) $$\mathrm{ETR}\;\left(\mathrm{bee}\right)=\frac{\mathrm{PEC}\;\left(\mathrm{bee}\right)}{\mathrm{NEC}\;\left(\mathrm{bee}\right)}$$

With,

- ETR_bee_ = Exposure Toxicity Ratio due to application
- PEC_bee_ = Exposure Rate = individual rate applied (g a.i./ha)
- NEC_bee_ = No Effect Rate for bees (g a.i./ha)

#### 2.2.3 Risk assessment on NTAs

Information from Table 1, Table 2, Table 3 was keyed into the PRIMET model, one after the other, except the column LD_50_Bees_ (μg/bee). For each active ingredient, the model estimated the Predicted Exposure Rate (PEC), Acceptable Effect Rate (AEC), and the Exposure Toxicity Ratio (ETR).

Standard species include two sensitive indicator species, the cereal aphid parasitoid *Aphidius rhopalosiphi,* and the predatory mite *Typhlodromus pyri*.

##### 2.2.3.1 Predicted exposure concentration (PEC)

Exposure in-field and off-field is estimated following Equations (4), (5)) respectively.

(4) PEC(in−field)=M∗MAF

(5) PEC(off−field)=M∗MAF∗(%drift/100/Veg)

With,

- PEC (in-field) = Exposure in-field (g a.i./ha)
- PEC (off-field) = Exposure off-field (g a.i./ha)
- M = Individual rate applied (g a.i./ha)
- MAF = Multiple Application Factor
- % drift = Percentage of drift spray (default value = 2.77 %)
− 100 = Factor to convert from % drift to drift factor
- Veg = Vegetation distribution factor (default value = 10)

The default value of the spray drift is 2.77 % [4]; this value considers a standard assessment at 1 m with a default value of 0.0277 [22].

The MAF depends on the number of application (*n*) and is presented in Table 4.

**Table 4:** MAF after n applications (default values for leaf dwelling arthropods).

*n* applications	1	2	3	4	5	6	7	8	>8
MAF after *n* applications	1.0	1.7	2.3	2.7	3.0	3.2	3.4	3.5	3.5

##### 2.2.3.2 “Safe” concentration

In the case of non-target arthropods, the Acceptable Effect Rate (AEC) was used instead of the No Effect Rate for effect assessment. It was computed following Equation (6).

(6) $$\mathrm{AE}C_{\mathrm{NTA}}=EF_{\mathrm{NTA}}\;*\;LR50_{\mathrm{NTA}}$$

With,

- AEC_NTA_ = Acceptable Effect Rate for Non-Target Arthropods (g a.i./ha)
- LR50_NTA_ = Dose that kills 50 % of *Typhlodromus pyri* or *Aphidius rhopalosiphi*, the most sensitive endpoint of the two organisms will be taken.
- EF_NTA_ = Extrapolation factor for effect assessment of Non-Target Arthropods

The extrapolation factor is centred on available semi-field data, where lethal, sub lethal and reproduction endpoints have been gauged for a considerable number of types of substances and species (The value of 2 is considered for risk assessment) [4].

##### 2.2.3.3 Risks to NTAs (ETR__NTA_)

a) In-_field_ risk to NTAs

The risk, expressed in Exposure Toxicity Ratio (ETR -*in-field*) because of applications was computed following Equation (7):

(7) $$\mathrm{ET}R_{\mathrm{NTA}}\;\left(\mathrm{in}-\mathrm{field}\right)=\mathrm{PE}C_{\mathrm{in}-\mathrm{field}}\;/\mathrm{AE}C_{\mathrm{NTA}}$$

With,

- ETR (in-field) = Exposure Toxicity Ratio in-field due to application
- PEC (in-field) = Exposure in-field (g a.i./ha)
- AEC_NTA_ = Acceptable Effect Rate to *Typhlodromus pyri* and *Aphidius rhopalosiphi* (g a.i./ha)
b) _Off_-field risks to NTAs

The risk, expressed in Exposure Toxicity Ratio (ETR-*off-field*) because of applications was evaluated following Equation (8):

(8) $$\mathrm{ET}R_{\mathrm{NTA}}\;\left(\mathrm{off}-\mathrm{field}\right)=\mathrm{PE}C_{\mathrm{off}-\mathrm{field}}\;/\mathrm{AE}C_{\mathrm{NTA}}$$

With,

- ETR (off-field) = Exposure Toxicity Ratio off-field due to application
- PEC (off-field) = Exposure off-field (g a.i./ha)
- AECNTA = Acceptable Effect Rate to *Typhlodromus pyri* and *Aphidius rhopalosiphi* (g a.i./ha)

### 2.3 Data processing and analysis

The risks to bees and NTAs (ETRs) were interpreted following Table 5.

**Table 5:**
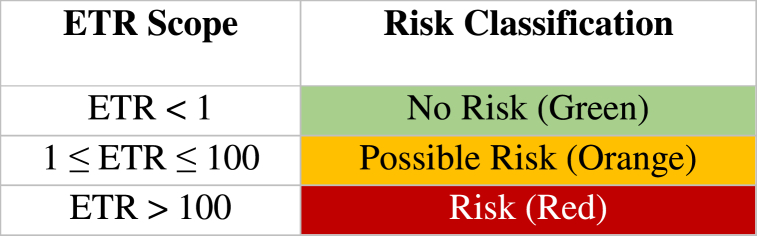
ETR scope, risk classifications.

Data was typed using Microsoft 365. The distribution of the ETRs according to pesticide families was plotted using SPSS version 21. A linear regression model was used to assess pesticide properties and usage scheme that may be predictor of risk.

## 3 Results

### 3.1 Various crops in the study area and pesticide categories used

**Fig. 2:**
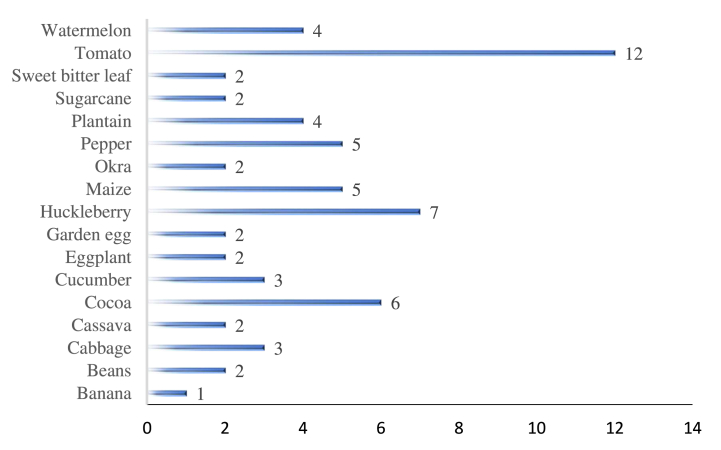
Crops cultivated by farmed in the study area during the survey period.

**Fig. 3:**
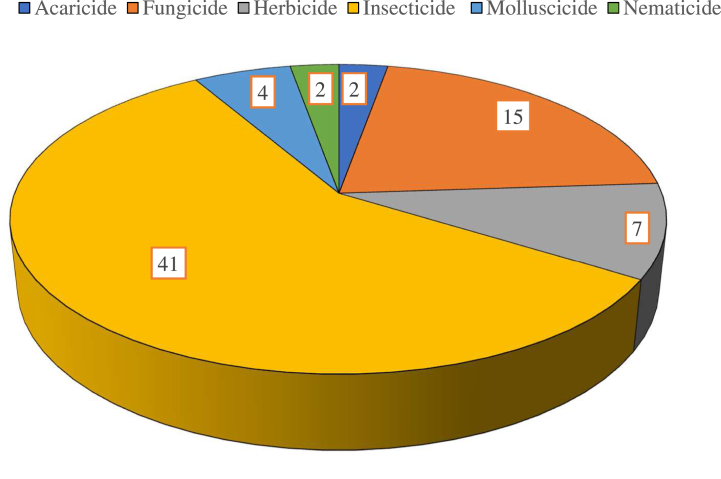
Pesticide categories in use in the study area.

Tomato was the main crop of the area (n = 12), followed by huckleberry (n = 7) as seen on Fig. 2. Insecticides (n = 41) and fungicides (n = 15) were the main pesticide families in use in the study area during the study period (Fig. 3).

### 3.2 Values of ETRs per pesticide and target organism

#### 3.2.1 Pesticide risks to bees

##### 3.2.1.1 The “no risk” category

The risk assessment to bees revealed that eight compounds were reported in the “*No Risk*” category. Carbendazim when used on tomato (ETR _bee_ = 0.96) and maize (ETR _bee_ = 0.19), mancozeb when applied on garden egg, pepper and cucumber (ETR _bee_ = 0.94), and also when applied on water melon (ETR _bee_ = 0.61) and tomato (ETR _bee_ = 0.59); copper oxide when used on cocoa (ETR _bee_ = 0.86), metalaxyl-M when applied on cucumber (ETR _bee_ = 0.82), cocoa (ETR _bee_ = 0.21 and ETR _bee_ = 0.12), diuron (ETR _bee_ = 0.69), chlorothalonil when applied on tomato (ETR _bee_ = 0.48), thiram when used on eggplant (ETR _bee_ = 0.26), metaldehyde when applied on huckleberry (ETR _bee_ = 0.02) (Table 6).

**Table 6:** Herbicides, acaricides, rodenticides, nematicides and molluscicides risk to bees and NTAs.

Pesticide	Category	Crops/Target	Risks to bees		Risks to NTAs		
			NEC_B_ee_ (g a.i./ha)	ETR_B_ee_	AEC__NTA_ (g a.i./ha)	ETR__NTA__(In-field)_	ETR__NTA__(Off-field)_
2,4-D	Herbicide	Weeds	4700	8.64	6000	11.5	0.032
Paraquat			453	4.42	20	100	0.28
				13.25		510	1.41
Diuron			4338	0.69	∗	∗	∗
Glyphosate			5000	1.8	8640	1.04	0.003
Nicosulfuron			1120	9.64	120	153	0.42
Paraquat dichloride			3600	1.94	∗	∗	∗
Abamectin	Acaricide	Tomato	0.05	20000	0.18	19440	53.9
		Beans				5556	15.4
Brodifacoum	Rodenticide	Maize, Cassava, Plantain	∗	∗	∗	∗	∗
		Tomato	∗	∗	∗	∗	∗
Ethoprophos	Nematicide	Plantain	203.5	14.74			
		Banana		157.2			
Metaldehyde	Molluscicide	Okra	4375	2.17	700	36.64	0.1
		Huckleberry		0.02		0.25	0.0007
		Plantain		3.09		44.36	0.12
		Watermelon		0.25		3.61	0.01

NEC_bee_ = No Effect Concentration for bees (g a.i./ha).

ETR_bee_ = Exposure Toxicity Ratio due to application.

AEC_NTA_ = Acceptable effect concentration for Non-Target Arthropods (g a.i./ha).

ETR (in- or off-field) = Exposure Toxicity Ratio due to application.

∗Risk evaluation not possible.

##### 3.2.1.2 The “possible risk” category

Pesticides predicted for a “Possible Risk” to bees included oxamyl, ethoprophos, paraquat, nicosulfuron, 2,4-D, copper oxide, metaldehyde, acetamiprid, paraquat dichloride, glyphosate, metalaxyl-M (Table 6, Table 7). The top position was occupied by oxamyl when used on watermelon (ETR _bee_ = 31.58) and tomato (ETR _bee_ = 26.32). Metalaxyl-M had the lowest ETR of this group when used on cabbage (ETR _bee_ = 1.23).

**Table 7:** Fungicide risks for bees and NTAs.

Fungicide	Crops	Risks to bees		Risks to NTAs		
		NEC__Bee_ (g a.i./ha)	ETR__Bee_	AEC_NTA_ (g a.i./ha)	ETR_NTA__(In-field)_	ETR_NTA__(Off-field)_
Carbendazim	Maize	2500	0.19	60	21	0.06
	Tomato		0.96		108	0.30
Chlorothalonil	Tomato	5050	0.48	524	12.37	0.03
Copper oxide	Cocoa	1100	0.86	52200	0.06	0.0002
	Tomato		3.64		0.18	0.0005
Mancozeb	Garden egg	4265	0.94	42.94	298	0.83
	Tomato		0.59		186	0.52
	Watermelon		0.61		139	0.39
	Pepper		0.94		326	0.90
	Cucumber		0.94		280	0.77
Mefenoxam (Metalaxyl-M)	Cocoa 1	4865	0.12	∗	∗	∗
	Cabbage		1.23			
	Cucumber		0.82			
	Cocoa 2		0.21			
Thiram	Eggplant	5000	0.26	1022	2.95	0.008

NEC_bee_ = No Effect Concentration for bees (g a.i./ha).

ETR_bee_ = Exposure Toxicity Ratio due to application.

AEC_NTA_ = Acceptable effect concentration for Non-Target Arthropods (g a.i./ha).

ETR (in- or off-field) = Exposure Toxicity Ratio due to application.

∗Risk evaluation not possible.

##### 3.2.1.3 “Risk” category

Compounds of the « *Risk* » category included abamectin, fipronil, imidacloprid, thiamethoxam, emamectin-benzoate, cypermethrin, lambda-cyhalothrin, indoxacarb and ethoprophos. The general tendency is that they were mainly insecticides (Table 8). Abamectin (acaricide) when applied on tomato and beans was the riskiest (ETR _bee_ = 20 000). Ethoprophos (nematicide) had the lowest ETR of the risk category when applied on banana (ETR _bee_ = 157.2).

**Table 8:** Insecticides risks for bees and others NTAs.

Insecticide	Crops	Bees		NTAs		
		NEC__Bee_ (g a.i./ha)	ETR__Bee_	AEC__NTA_ (g a.i./ha)	ETR__NTA_IF_	ETR__NTA_OF_
Acetamiprid	Tomato	404.5	2.5	4	750	2.08
	Huckleberry		2.2		608	1.68
Chlorpyrifos-ethyl	Eggplant	3.4 3.4	309	0.4	6038	16.72
	Pepper		353		8100	22.44
Cypermethrin	Maize	1.15	522	0.006	237 900	659
	Cucumber		783		356 900	989
	Plantain		870		396 600	1098
	Tomato		870		465 500	1289
	Huckleberry		870		551 700	1528
Emamectin-benzoate	Sweet bitter leaf	0.18	1944	1	350	0.97
	Cassava		5000		1530	4.24
	Sugarcane		2778		1750	4.85
Fipronil	Plantain	0.295	4237	0.02	187 500	519
	Garden egg		2712		128 000	355
	Cabbage		3105		121 500	337
	Pepper		2373		94 500	262
	Cassava		4766		189 800	526
	Watermelon		2373		805 00	223
	Okra		8475		212 500	589
	Maize		6780		170 000	471
	Sugarcane		4068		180 000	499
	Cocoa		6356		281 300	779
	Tomato		1356		64 000	177
Imidacloprid	Huckleberry	0.185	2703	0.044	19 320	54
	Tomato		7568		111 400	309
	Cocoa		4865		65 450	181
	Pepper		5405		52 270	145
Indoxacarb	Huckleberry	4	225	10.2	238	0.66
Lambda-cyhalothrin	Huckleberry	1.9	526	0.0034	941 200	2607
	Tomato		684		1 147 000	3177
	Maize		368		350 000	970
	Pepper		526		676 500	1874
Oxamyl	Beans	19	211	0.06	113 300	314
	Tomato		26.32		8333	23
	Sweet bitter leaf		211		66 670	185
	Huckleberry		211		113 300	314
	Watermelon		31.58		17 000	47
Thiamethoxam	Cocoa	0.25	2520	0.26	5573	15.44
	Cabbage		2000		4423	12.3
	Maize		3600		9346	26
	Tomato		6000		13270	37

NEC_bee_ = No Effect Concentration for bees (g a.i./ha).

ETR_bee_ = Exposure Toxicity Ratio due to application.

AEC_NTA_ = Acceptable effect concentration for Non-Target Arthropods (g a.i./ha).

ETR (in- or off-field) = Exposure Toxicity Ratio due to application.

#### 3.2.2 Pesticide risks to NTAs

##### 3.2.2.1 “No risk” to NTAs category

a In-field risk

Metaldehyde (Table 6), when applied on huckleberry was predicted for “No Risk” for NTAs in-field (ETR _IF_ = 0.25). Moreover, when used on tomato (0.18) and cocoa (0.06), copper oxide was predicted to be “no risk” (Table 7).

b _Off_-field risk

Pesticide compounds predicted for “no off-field risk” to NTAs included emamectin-benzoate when applied on sweet bitter leaf (ETR_OF_ = 0.97), mancozeb when used on pepper, cucumber, garden egg and tomato, nicosulfuron (ETR_OF_ = 0.42), carbendazim when used on tomato and maize, paraquat (ETR_OF_ = 0.28), metaldehyde (applied on huckleberry, plantain and okra), chlorothalonil (ETR_OF_ = 0.3 when used on tomato), 2,4-D, thiram (ETR_OF_ = 0.008 when applied on eggplant), glyphosate (ETR_OF_ = 0.003) and copper oxide (applied on tomato and cocoa) (Table 6, Table 7, Table 8).

##### 3.2.2.2 “Possible risk” to NTAs category

i) In-_field_

Paraquat, metaldehyde, carbendazim, chlorothalonil, 2,4-D, thiram and glyphosate were the compounds predicted for this category. Paraquat had the highest ETR (100), followed by metaldehyde (when used on plantain and okra); the bottom was occupied by glyphosate (ETR_IF_ = 1.04) (Table 6).

ii) Possible Off-field risk to NTAs

When applied on tomato, abamectin (acaricide) modelled a possible off-field risk to NTAs (ETR_OF_ = 53.9). Other compounds of this category included imidacloprid, oxamyl, thiamethoxam, chlorpyrifos-ethyl, emamectin-benzoate, acetamiprid and paraquat (Table 6, Table 7, Table 8).

##### 3.2.2.3 “Risk” to NTAs category

a. In-field risk to NTAs

Compounds that were predicted to pose “Risk” to NTAs in-field included: lambda-cyhalothrin, cypermethrin, fipronil, oxamyl, imidacloprid, abamectin, thiamethoxam, chlorpyrifos-ethyl, emamectin-benzoate, acetamiprid, paraquat, mancozeb, indoxacarb, nicosulfuron and carbendazim. Lambda-cyhalothrin occupied the topmost position when applied on tomato (ETR_IF_ = 1 147 000), huckleberry (ETR_IF_ = 941 200) and pepper (ETR_IF_ = 676 500) (Table 8).

b. Off-_field_ risk to NTAs

Pesticides that were predicted for a certain off-field risk to NTAs were all from the insecticide family. The top position was occupied by lambda-cyhalothrin when applied on tomato (ETR_OF_ = 3177). Other compounds included cypermethrin, fipronil, oxamyl and imidacloprid (Table 8).

### 3.3 Distribution of ETRs according to pesticide categories for bees and others NTAs

**Fig. 4:**
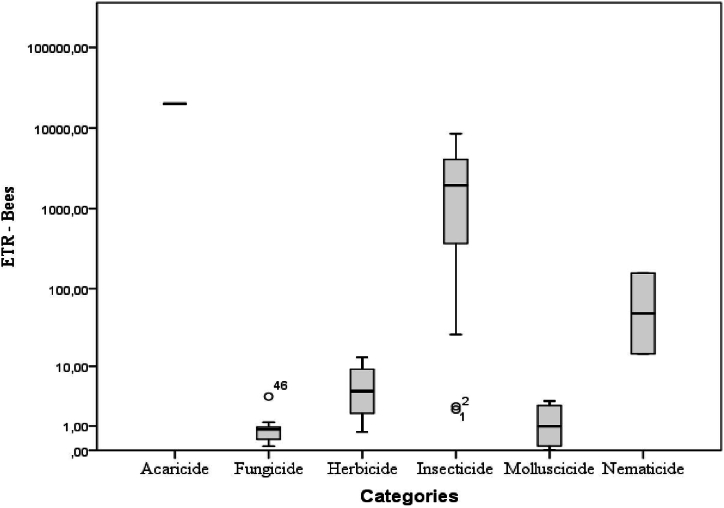
ETRs according to pesticide categories for bees.

**Fig. 5:**
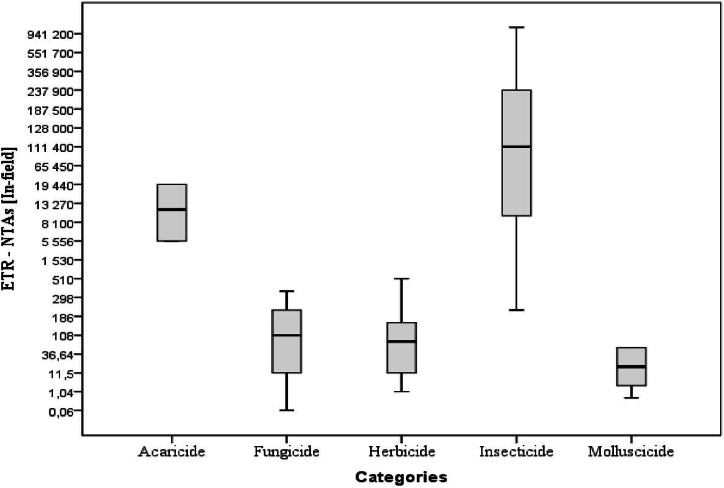
ETRs according to pesticide categories for NTAs (In-field).

**Fig. 6:**
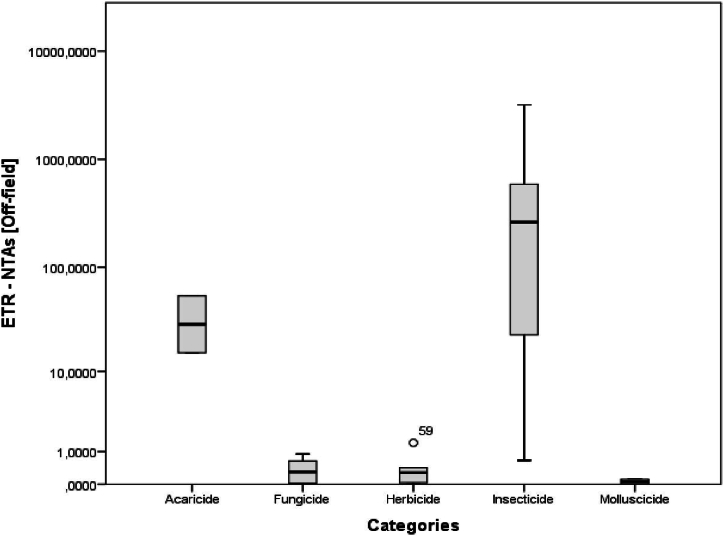
ETRs according to pesticide categories for NTAs (Off-field).

Fig. 4, Fig. 5, Fig. 6 give evidence of a high risk associated with insecticide usage as compared to other pesticide categories. The ETRs of insecticides were generally high both for bees and NTAs.

### 3.4 Relationship between pesticide usage scheme and ecotoxicological property, and risk

For the risk assessment to bees, the linear regression model revealed that the toxicity of a compound was a significant predictor of risk, whatever the rate of application, the number of applications per crop seasons and the time interval between applications. Moreover, the number of applications er cropping cycle was positively linked to the ETR_bee._ (Equation (9)).

(9) ETR_bee_ = 1355 + 21.07 [Application Interval] - 0.04 [Rate of Application] + 272 [Number of Applications] - 32.67 LD50_bee_

The risk assessment to NTAs infield revealed that the rate of application and the number of applications per crop season were positively associated with the ETR_IF_ (NTAs). The association was negative between ETR_IF_ (NTAs) and the application interval as well as the LR50 (Equation (10)).

(10) ETR_IF_ (NTAs) = 198268 - 2102 [Application Interval] + 21 [Rate of Application] + 3629 [Number of Applications] - 8507 LR50_NTAs_

The ability of pesticides to pose off-field risk to NTAs (ETR_OF_) was positively associated with the number of applications per crop season. The association was negative and weak between and application interval, as well as the LR50_NTAs_ (Equation (11)).

(11) ETR_OF_ (NTAs) = 355–2.17 [Application Interval] - 0.013 [Rate of Application] + 22 [Number of Applications] - 0.015 _LR50NTAs_

## 4 Discussion

### 4.1 Pesticide usage and crop production in the study area

This research evaluated the ecological risk of synthetic pesticides frequently used by farmers at the southeast slopes of Mount Cameroon on pollinators (bees) and other NTAs (represented by two sensitive species *A. rhopalosiphi* and *T. pyri*) using the *Pesticide RIsk in the tropics to Man, Environment and Trade* (PRIMET) model. Insecticides were the most used compounds in the area, followed by fungicides and herbicides; these same findings have previously been documented in Cameroon [13,15]. This suggests that insects and fungi represent serious threat to crop production in the study area. The availability of a huge diversity of synthetic pesticides homologated in Cameroon [23] may also explain these findings. Moreover, tomato and huckleberry were the most common crops during the study. Tomatoes are vulnerable to many infections and common symptoms observed in Buea include leaf curl, leaf roll, general chlorosis and shoe stringing [24], hence the use of many pesticides. Described to be the most highly cultivated fruity plant in Cameroon [25], the production of tomato (*Solanum lycopersicum*) has been reported to be a main source of revenue for youths in the Buea metropolis [26].

### 4.2 Ecological risk assessment of pesticides

#### 4.2.1 Pesticide risks to bees

Carbendazim, mancozeb, copper oxide (when applied on cocoa), metalaxyl-M (applied on cocoa and cucumber), diuron, chlorothalonil, thiram and metaldehyde were the pesticide active ingredients predicted for “No Risk” to bees under the prevailing usage scheme. The fungicide ccarbendazim has been reported to cause no acute death of bees and does not disturb their gut microbiome [27]. The results on diuron confirm previous reported according to which diuron (NEC = 4338 g a.i./ha; ETR_bee_ = 0.69) modelled no risk to bees [8,16]. These relatively safe compounds are mainly fungicides, herbicides and molluscicides (metaldehyde). The case of metaldehyde should be interpreted with care as it was predicted for “no risk” when used on huckleberry and watermelon; its use on other crops is not without risk.

In the “possible risk” category, eleven (11) compounds were recorded: oxamyl, ethoprophos, paraquat, nicosulfuron, 2,4-D, copper oxide (when applied on tomato), metaldehyde, acetamiprid, paraquat dichloride, glyphosate and metalaxyl-M (when used on cabbage). In this group metaldehyde overlaps with the “no risk” category; this molluscicide was reported to have a “possible risk” to bees when applied on okra and plantain. Moreover, among these pesticides, acetamiprid was predicted to pose a possible risk to bees in the Tiko municipality with an ETR of 12 in a previous report [16].

Nine (09) compounds were predicted to pose a “definite risk” to bees namely abamectin, fipronil, imidacloprid, thiamethoxam, emamectin-benzoate, cypermethrin, lambda-cyhalothrin, indoxacarb and ethoprophos. Among these compounds, cypermethrin and imidacloprid, have previously been reported to model very high risk to bees in the Western Highlands Agroecological Zone of Cameroon [28]. Moreover, three of these pesticides namely fipronil, cypermethrin and imidacloprid were reported to model risk to bees in the Tiko plain in the Cameroon coastal area [16]. Used on tomato and beans, the acaricide abamectin had the highest ETR_bee_ and was the compound with the highest ETR_bee_ in this study. With a high affinity to soil particles [29], abamectin compound has been reported to be very persistent in soil and food products [30], this may account for its ability to model high risks. In general, the main drivers of pesticide risk to bee include environmental persistence, pesticide mixture and spray drift. Spray drift is a function of meteorology, spray pressure and spray nozzles [31,32].

#### 4.2.2 Pesticide risks to others NTAs

Metaldehyde (molluscicide) and copper oxide (fungicide) were predicted by PRIMET to pose no in-field risk to NTAs. These two pesticides also posed no risks to bees under the prevailing application scheme. In bioassays, copper has been reported to be “harmless” to beneficial non-target arthropods such as *Chrysoperla externa* and *Coleomegilla quadrifascia* [33]*.* In this study, it was used 3 times per crop season on tomato and 6 times per season on cocoa; it generally has low toxicity towards NTAs (LR_50_ = 26100 g a.i./ha). Metaldehyde is a contact and systemic molluscicide bait for managing slugs and snails [21]; many of its formulations are designed to be less toxic to non-target organisms.

For the “no off-field risk” to NTAs, 11 compounds were registered: emamectin-benzoate, mancozeb, nicosulfuron, carbendazim, paraquat, metaldehyde, chlorothalonil, 2,4-D, thiram, glyphosate, and copper oxide. Many of them (91 %) are not insecticides, assuming that they are formulations designed to be less harmful on non-target arthropods when applied in recommended doses. Emamectin-benzoate was the only insecticide predicted for “no off-field risk”; this compound has been reported to have low risk to natural predator such as *Harmonia axyridis* (Coleoptera: Coccinellidae) [34].

Seven (07) pesticides (paraquat, metaldehyde, carbendazim, chlorothalonil, 2,4-D, thiram and glyphosate) were reported to pose a “possible in-field risk” to NTAs. No insecticide was recorded in this category. In the “possible off-field risk” to NTAs, eight (08) pesticides **(**abamectin, imidacloprid, oxamyl, thiamethoxam, chlorpyrifos-ethyl, emamectin-benzoate, acetamiprid and paraquat) were recorded. Mitigations strategies should be implemented for these compounds so that they don't reach the “risk” category.

Fifteen (15) active ingredients were predicted by the model to pose a certain in-field risk to NTAs. Those compounds included lambda-cyhalothrin, cypermethrin, fipronil, oxamyl, imidacloprid, abamectin, thiamethoxam, chlorpyrifos-ethyl, emamectin-benzoate, acetamiprid, paraquat, mancozeb, indoxacarb, nicosulfuron and carbendazim. Most of these compounds have previously been reported to model unacceptable risk to non-target natural predator *Harmonia axyridis* (Coleoptera: Coccinellidae) [34]. Contrary to our current report, in a previous ecological risks assessment, thiamethoxam was reported to pose “no risk” to NTAs in the monomodal equatorial agroecological zone of Cameroon [8]. Thiamethoxam (neonicotinoid) exhibits outstanding insecticidal activity by interfering with the nicotinic acetylcholine receptor in insects [35].

In the “definite off-field risk” to NTAs category, five (05) compounds, all insecticides (lambda-cyhalothrin, cypermethrin, fipronil, oxamyl and imidacloprid) were registered. Among these five compounds, fipronil was earlier reported to pose “risk” to NTAs with ETRs of 14 080 and 39, respectively for in-field and off-field risks [8]. The insecticide fipronil has been reported to significantly reduced the population of soil non-target arthropods and proposals have been made for its substitution with less hazardous pesticides [36] as its use is not without impact on local arthropods species [37]. Previous reports have also documented the adverse effects of imidacloprid: it was reported to have the greatest harmful effects on the arthropod community in terms of species diversity, species composition and natural enemy density as compared to other insecticides [38].

The lack of ecotoxicological data on pesticides such as mefenoxam or metalaxyl - M (fungicide), paraquat dichloride (herbicide), diuron (herbicide), brodifacoum (rodenticide) and ethoprophos (nematicide - insecticide) limited the EcoRA of these compounds.

#### 4.2.3 Risk potential according to pesticide categories

There was evidence that insecticides were of more risk to beneficial arthropods, as compared to other types of pesticides. This same distribution has been previously reported in ecological risk assessment of pesticides [8,16,28,39], a confirmation of the high risks related to insecticide usage in agriculture. This finding is obvious since insecticides are designed for insects, nevertheless, the risk related to other pesticide families should not be underrated. The side effects of insecticides on arthropods is varied: insecticides have been reported to have effect on cognitive functions, behaviour and physiology of bees [40]. Moreover, it has been documented that neurotoxic insecticides are able to affect the cognitive abilities of bees, impairing their performance and ultimately impacting on the viability of the colonies [41].

Fungicides had generally low risks to bees and NTAs; similar findings were reported in previous risks assessment where the majority of fungicides posed low to no risks to NTAs and bees in the Tiko municipality [8,16]. Studies on fungicides’ impacts on bees remain poorly documented in Africa [42]. Previous reports stipulate that fungicides are generally less risky to arthropods as compared to insecticides [43]. Nevertheless, they should be used with care because their behaviour in the presence of other compounds is unpredictable. For instance, in the presence of insecticides, they have synergistic effects [44]. Therefore, using fungicides in mixtures with other pesticides, a common practice exhibited by many pesticide users as documented by previous surveys in Cameroon [13,15], should be discouraged.

As earlier reported in the Cameroon coastal zone [8,16], herbicides risk to bees and NTAs was low in the present study. Herbicide toxicity to bees has been reported to be very low [45]. Studies on herbicides effects on bees remain rare in Africa [42]; this may be related to their relatively low toxicity to bees.

### 4.3 Pesticide legality and registration process in Cameroon

The registration process of pesticides in Cameroon is governed by two main bodies: the National Commission of Pesticide Registration and Certification of Sprayers and the Ministry of Agriculture & Rural Development (MINADER). The registration process involves an initial assessment, the determination of the suitability of the compound in Cameroon and surveillance [46]. Unfortunately, the national capacity and ability to evaluate the pesticide risk on human health and the environment grounded on national data is inadequate [47]; as a result, pesticide ecotoxicological data remains poorly documented mainly because of lack of facilities (GC-MS, LC-MS, etc …) and inadequate collaboration between researchers and those regulatory bodies.

## 5 Conclusion

Synthetic pesticide usage in agriculture may help to increase the yield but is risky to bees, the main pollinators of many tropical crops, and other non-target arthropods (NTAs). Pesticides active ingredients such as abamectin, fipronil, imidacloprid, thiamethoxam, emamectin-benzoate, cypermethrin and lambda-cyhalothrin that were predicted to pose “Risk” both to bees and others NTAs require serious risk mitigation strategies for their use. Mitigation strategies include the education of farmers on the role of biodiversity and risks of overusing synthetic pesticides, creation of buffer zones around plantations to allow beneficial insects, using selective pesticides. Despite being a case study with the PRIMET model, our findings provide an update on pesticide ecotoxicology at the regional level and under the tropics. The implementation of precision agriculture with IPM is an important step to avoid pesticide dependence. Based on our findings, we recommend the inclusion of the PRIMET model as a tool of the National Registration Commission of Phytosanitary Products and Certification of Sprayers of the Ministry of Agriculture and Rural Development (MINADER) of the Republic of Cameroon, as well as the creation and implementation of a synthetic pesticide reduction programme in Cameroon.

## CRediT authorship contribution statement

**Daniel Brice Nkontcheu Kenko:** Writing – original draft, Supervision, Methodology, Formal analysis, Conceptualization. **Agathe Lambou Fotio:** Writing – review & editing, Supervision. **Kenfack Donhachi Aimerance:** Writing – review & editing, Conceptualization. **Derick Atemlefac Acha:** Methodology, Investigation, Funding acquisition. **Miranda Egbe Awo:** Writing – review & editing, Conceptualization. **Eric Bertrand Fokam:** Supervision, Project administration, Conceptualization.

## Data availability statement

Data will be made available upon reasonable request to the corresponding author.

## Declaration of competing interest

The authors declare that they have no known competing financial interests or personal relationships that could have appeared to influence the work reported in this paper.
